# Comparative associations of the Advanced Lung Cancer Inflammation Index and Prognostic Nutritional Index with osteoporosis among adults in the United States: A cross-sectional analysis

**DOI:** 10.1097/MD.0000000000049531

**Published:** 2026-07-17

**Authors:** Pujian Zhong, Chaoyi Yin, Binshan Zhang, Yiqi Wu, Canwei Hu, Zhiqing Chen, Wenren Wu, Zhuoqin Pan

**Affiliations:** a Dongguan Hospital of Guangzhou University of Chinese Medicine, Dongguan, Guangdong, China.

**Keywords:** bone mineral density, calibration-based balancing weights, nutritional status, osteoporosis, systemic inflammation

## Abstract

Osteoporosis is a major public health problem in aging populations and is increasingly linked to interactions between nutritional status and systemic inflammation. The Prognostic Nutritional Index (PNI) and the Advanced Lung Cancer Inflammation Index (ALI) are established immunonutritional markers in oncology, but their associations with osteoporosis in the general population remain unclear. We examined the cross-sectional associations of PNI and ALI with osteoporosis and compared the consistency of these associations across analytic strategies. We conducted a cross-sectional analysis of adults from the National Health and Nutrition Examination Survey 1999–2018. A total of 18,497 participants with valid dual-energy X-ray absorptiometry-derived bone mineral density data and complete components for PNI and ALI calculation were included. Osteoporosis was defined as a femoral neck or lumbar spine *T*-score ≤ −2.5. Survey-weighted multivariable logistic regression models were used to estimate odds ratios (ORs) and 95% confidence intervals (CIs). Sensitivity analyses using calibration-based balancing weights and restricted cubic splines were performed. In fully adjusted models, ALI showed a consistent inverse association with prevalent osteoporosis. Participants in the highest ALI quartile had significantly lower odds of osteoporosis than those in the lowest quartile (OR 0.590, 95% CI 0.447–0.778; *P* < .001), and this association remained significant in sensitivity analyses (OR 0.79, 95% CI 0.64–0.97; *P* = .027). PNI was inversely associated with osteoporosis in continuous analyses (OR 0.782 per 1-SD increase; *P* = .011), but this association was attenuated in categorical analyses and not significant in sensitivity analyses. Dose–response analyses suggested an approximately linear inverse relationship for ALI, whereas results for PNI were less consistent. Both PNI and ALI were associated with osteoporosis in US adults, but ALI showed a more consistent inverse association across analytical strategies. As a composite index integrating body mass index, albumin, and the neutrophil-to-lymphocyte ratio, ALI may warrant further evaluation as a practical composite marker associated with bone health. Given the cross-sectional design, these findings should be interpreted as associational rather than causal.

**Key points**

● Comparative evaluation of Prognostic Nutritional Index and Advanced Lung Cancer Inflammation Index (ALI) in relation to osteoporosis in a large, nationally representative adult sample.
● ALI showed a more consistent inverse association with osteoporosis than Prognostic Nutritional Index across adjusted models and sensitivity analyses.
● Calibration-based balancing weights were used as a robustness check to reduce covariate imbalance and support sensitivity analyses.
● Restricted cubic splines suggested an approximately linear dose–response pattern for ALI.
● Findings support an osteoimmunology framework linking immunonutritional status to skeletal health.

## 1. Introduction

The rapid global shift toward population aging is accompanied by a rising burden of osteoporosis,^[1]^ a systemic skeletal disorder characterized by low bone mass and microarchitectural deterioration that increases fracture risk.^[2,3]^ Because bone loss is often clinically silent until a fragility fracture occurs, practical and noninvasive markers that can help identify higher-risk individuals are needed.^[4,5]^ Although dual-energy X-ray absorptiometry (DXA) is the diagnostic reference standard, its availability is uneven, and bone mineral density (BMD) alone provides limited information on systemic processes that may accelerate bone loss.^[6]^

Beyond endocrine and calcium-centric explanations, osteoporosis is increasingly viewed through an osteoimmunology lens, in which immune dysregulation and chronic low-grade inflammation contribute to an imbalance in bone remodeling.^[7,8]^ Aging-related inflammatory activation can favor osteoclastogenesis and bone resorption,^[9,10]^ while compromised nutritional status may impair bone formation by limiting substrate supply and weakening anabolic signaling.^[11]^ In real-world populations, inflammation and malnutrition frequently coexist, making integrative immunonutritional indicators attractive for population-level health assessment.^[12,13]^

The Prognostic Nutritional Index (PNI), derived from serum albumin and lymphocyte count, has been widely used to reflect immunonutritional reserve across multiple clinical settings.^[14,15]^ The Advanced Lung Cancer Inflammation Index (ALI) extends this concept by integrating body mass index (BMI), albumin, and the neutrophil-to-lymphocyte ratio (ALI = BMI × albumin/NLR), capturing anthropometric reserve, nutritional status, and inflammatory balance in a single metric.^[16,17]^ Given the established links of low body mass and inflammatory predominance with poorer skeletal outcomes, ALI may provide a complementary perspective to PNI^[18,19]^; however, because BMI is embedded in ALI, careful adjustment and sensitivity analyses are essential for interpretation.^[20,21]^

Evidence directly comparing PNI and ALI in relation to osteoporosis in the general population remains limited, and prior studies often lacked large representative samples or rigorous strategies to address confounding. Using data from the National Health and Nutrition Examination Survey (NHANES) 1999–2018, we evaluated the associations of PNI and ALI with osteoporosis prevalence. In addition to conventional survey-weighted multivariable regression, we applied calibration-based balancing weights (CBW) as a robustness-oriented sensitivity approach to improve covariate balance across exposure strata. We hypothesized that both indices would be associated with osteoporosis, while allowing for possible differences in the consistency of associations across analytic strategies.

## 2. Methods

### 2.1. Data source and study design

We used data from the NHANES, an ongoing program designed to evaluate the health and nutritional status of the noninstitutionalized civilian US population through standardized interviews and examinations. NHANES applies a complex, stratified, multistage probability sampling design, enabling nationally representative estimates when appropriate weights, strata, and primary sampling units are incorporated. For this analysis, we pooled NHANES cycles from 1999–2018; bone outcomes based on DXA were available only in specific examination cycles (2005–2010, 2013–2014, and 2017–2018), which therefore determined the analytic sample. NHANES protocols were approved by the National Center for Health Statistics Research Ethics Review Board, and all participants provided written informed consent.

### 2.2. Study population

Among 101,316 participants enrolled in NHANES 1999–2018, we restricted analyses to adults (≥20 years). We then limited the sample to cycles with DXA data and excluded participants lacking valid femur and lumbar spine DXA measurements. Participants missing any component required to compute the immunonutritional indices (albumin, neutrophils, lymphocytes, or BMI) were also excluded. For remaining covariates not required for index construction, the primary regression analyses used a complete-case approach; participants with missing values in covariates required for a given model were excluded from that specific analysis, and no multiple imputation was performed. The final analytic sample included 18,497 adults (participant selection is summarized in the study flow diagram).

### 2.3. Exposure variables: immunonutritional indices

The primary exposures were the PNI and the ALI, calculated from examination-based anthropometrics and laboratory measurements obtained using standardized NHANES protocols.

PNI was calculated as:


$$\begin{array}{cc} PNI= & 10\times Albumin\;(g/dL)\\ & +0.005\times Total\;lymphocyte\;count\;(/\mu L) \end{array}$$

ALI was calculated as:


$$\begin{array}{c} ALI=\frac{BMI\;(kg/m^{2})\times Albumin\;(g/dL)}{NLR} \end{array}$$

where NLR is the absolute neutrophil count divided by the absolute lymphocyte count.

Both indices were analyzed as continuous variables scaled per 1-standard deviation (SD) increase (z-score) to facilitate comparison of effect sizes and as quartiles (Q1 as reference) to assess potential dose–response patterns.

### 2.4. Outcome variable: osteoporosis

The primary outcome was osteoporosis defined using DXA-derived BMD at the femoral neck and lumbar spine. NHANES DXA measurements followed centralized quality assurance procedures, including ongoing phantom scanning and monitoring. Osteoporosis was defined as a *T*-score ≤ −2.5 at either the femoral neck or lumbar spine. *T*-scores were calculated using the NHANES III reference approach (White women aged 20–29 years) as commonly applied in NHANES-based osteoporosis research.

### 2.5. Covariates

Covariates were selected a priori based on established osteoporosis risk factors and potential confounding of immunonutritional–bone associations. These included: age, sex, race/ethnicity, education level, poverty–income ratio (PIR), marital status, smoking status, alcohol consumption, diabetes, hypertension, and serum creatinine.

Age was analyzed as a continuous variable. Sex was categorized as male or female. Race/ethnicity was classified as non-Hispanic White, non-Hispanic Black, Hispanic, and other race/ethnicity. Education level was categorized as less than high school, high school graduate/GED, and more than high school. PIR was analyzed as a continuous socioeconomic indicator. Marital status was categorized as married or living with a partner versus not married. Smoking status was classified as never smoker, former smoker, and current smoker. Alcohol consumption status was classified as never drinker, former drinker, and current drinker. Diabetes and hypertension were included as binary covariates. Serum creatinine was analyzed as a continuous laboratory covariate.

### 2.6. Statistical analysis

All analyses incorporated NHANES sampling weights, strata, and primary sampling units to account for the complex survey design and to produce nationally representative estimates. For pooled cycles, we constructed multi-cycle Mobile Examination Center examination weights according to NHANES guidance by dividing the 2-year Mobile Examination Center weights by the number of 2-year cycles contributing DXA data to this analysis.

Participant characteristics were summarized by osteoporosis status using weighted means (standard errors) for continuous variables and weighted proportions for categorical variables. Between-group differences were assessed using design-based tests (design-based Kruskal–Wallis tests for continuous variables), as appropriate for complex survey data.

We then fitted survey-weighted logistic regression models to estimate odds ratios (ORs) and 95% confidence intervals (CIs) for osteoporosis in relation to PNI and ALI. Models were specified sequentially: an unadjusted model; a demographic-adjusted model (age, sex, and race/ethnicity); and a fully adjusted model further including socioeconomic factors, lifestyle factors, comorbidities, and relevant anthropometric/laboratory covariates. Because BMI is a component of the ALI formula, BMI was included as a covariate in PNI models but was not included in ALI models to avoid component adjustment and collinearity.

To evaluate robustness to covariate imbalance and potential residual confounding, we conducted a sensitivity analysis using CBW. In brief, calibration-based weighting reweights the sample to improve balance of prespecified covariate moments across exposure strata. Compared with conventional propensity-score weighting, this approach directly targets prespecified balance constraints and was used here as a robustness-oriented sensitivity analysis rather than as the primary analytic framework. For these CBW analyses only, each index was dichotomized at the survey-weighted median to define prespecified exposure strata. This binary specification was used to facilitate stable calibration and to create broadly comparable groups for a robustness-oriented balancing analysis, rather than as the primary exposure modeling strategy. We acknowledge that dichotomization may reduce information and statistical power; therefore, continuous and quartile-based analyses remained the primary analyses. We estimated effects using outcome models fit with the calibrated weights and additionally reported an augmented specification combining CBW with outcome-model covariate adjustment as a robustness check.

As an additional sensitivity analysis, we conducted prespecified scenario-based cross-sectional models on the prevalence-ratio (PR) scale using survey-weighted generalized linear models with a log link. These PR models were included as a complementary effect-scale robustness check, because PRs are often more directly interpretable than ORs in cross-sectional settings, whereas survey-weighted logistic regression remained the primary analytic framework for the main analyses and spline models. Potential nonlinear dose–response relationships were examined using restricted cubic splines. Prespecified subgroup analyses were conducted by sex, race/ethnicity, smoking, alcohol use, diabetes, and hypertension; effect modification was assessed by including multiplicative interaction terms. All analyses were conducted in R using the survey analysis framework, and a two-sided *P* value < .05 was considered statistically significant.

## 3. Results

### 3.1. Baseline characteristics of the study population

**Figure 1.**
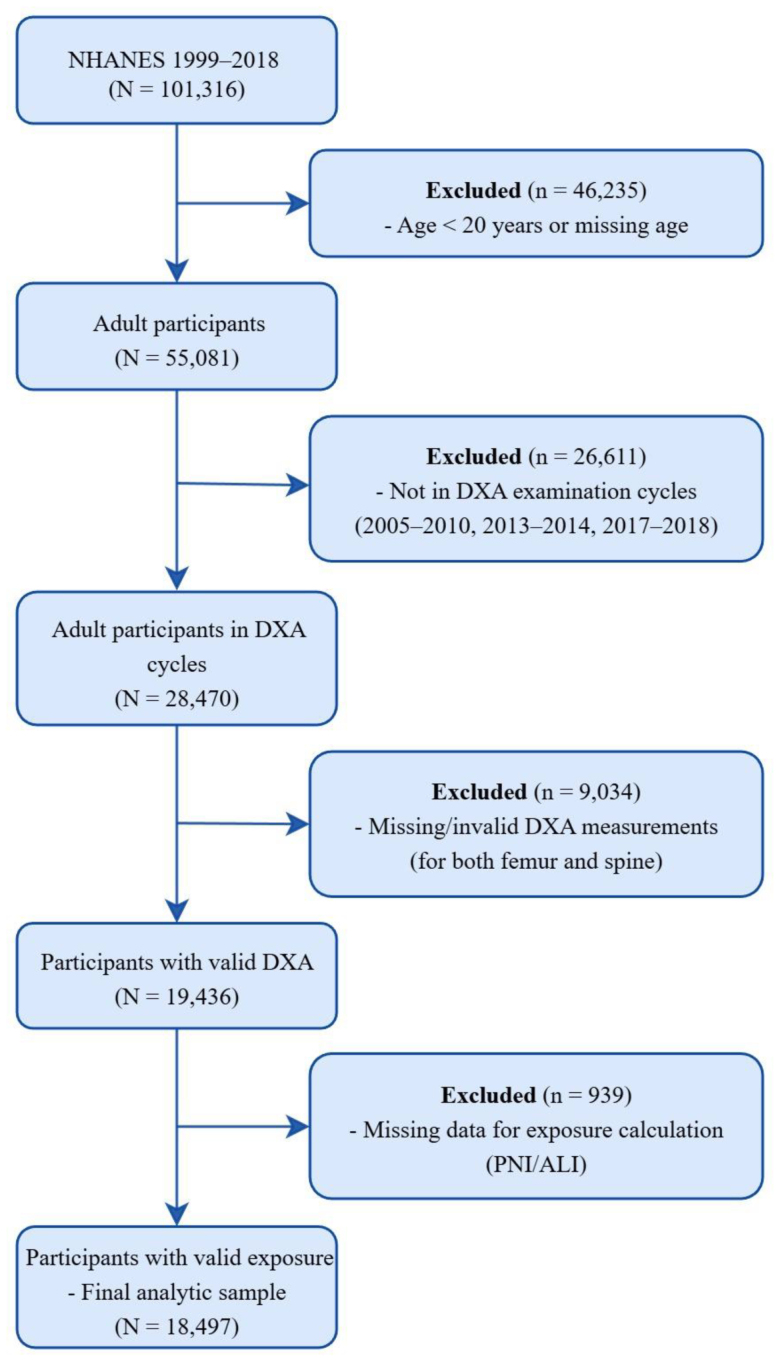
Flow diagram of participant selection. ALI = Advanced Lung Cancer Inflammation Index, DXA = dual-energy X-ray absorptiometry, NHANES = National Health and Nutrition Examination Survey, PNI = Prognostic Nutritional Index.

The derivation of the analytic sample is shown in Figure 1, andbaseline characteristics are summarized in Table 1. In the weighted population, participants with osteoporosis were markedly older than those without osteoporosis (64.97 vs 46.02 years, *P* < .001) and were predominantly female (78% vs 49%, *P* < .001). Osteoporosis was also more frequent among non-Hispanic White participants and among individuals with lower educational attainment and lower PIR (all *P* < .001).

**Table 1:** Characteristics by osteoporosis status (weighted).

Characteristic	Overall N = 13,468†	No N = 12,099†	Yes N = 1369†	*P*-value*
Age, yrs	47.66 (15.69)	46.02 (14.89)	64.97 (13.32)	<.001
Sex				<.001
Male	6644 (49%)	6314 (51%)	330 (22%)	
Female	6824 (51%)	5785 (49%)	1039 (78%)	
Race/ethnicity				<.001
Non-Hispanic White	6135 (69%)	5411 (69%)	724 (75%)	
Non-Hispanic Black	2502 (10%)	2391 (10%)	111 (4%)	
Hispanic	3757 (14%)	3397 (14%)	360 (11%)	
Other race/ethnicity	1074 (7%)	900 (7%)	174 (10%)	
Education level				<.001
More than high school	6787 (59%)	6232 (60%)	555 (50%)	
High school graduate/GED	3125 (24%)	2789 (24%)	336 (26%)	
Less than high school	3544 (17%)	3070 (16%)	474 (24%)	
Poverty-income ratio (PIR)	3.12 (1.63)	3.16 (1.63)	2.72 (1.56)	<.001
Marital status				<.001
Married or living with partner	8394 (66%)	7705 (67%)	689 (53%)	
Not married	5069 (34%)	4390 (33%)	679 (47%)	
Body mass index (BMI), kg/m^2^	27.80 (5.62)	28.06 (5.60)	25.12 (5.09)	<.001
Waist circumference, cm	96.23 (14.46)	96.73 (14.42)	90.87 (13.81)	<.001
Smoking status				.347
Never smoker	7361 (55%)	6570 (54%)	791 (55%)	
Former smoker	3169 (24%)	2826 (24%)	343 (25%)	
Current smoker	2933 (22%)	2699 (22%)	234 (20%)	
Alcohol consumption status				<.001
Never drinker	3345 (21%)	2826 (20%)	519 (33%)	
Former drinker	1592 (10%)	1341 (9%)	251 (18%)	
Current drinker	7882 (69%)	7363 (70%)	519 (49%)	
Diabetes mellitus	1458 (8%)	1248 (7%)	210 (11%)	<.001
Hypertension	4402 (29%)	3769 (28%)	633 (42%)	<.001
Serum creatinine, mg/dL	0.89 (0.34)	0.89 (0.32)	0.91 (0.49)	<.001
HbA1c, %	5.55 (0.86)	5.54 (0.87)	5.72 (0.81)	<.001
Prognostic Nutritional Index (PNI)	53.34 (9.73)	53.50 (10.04)	51.65 (5.27)	<.001
Advanced Lung Cancer Inflammation Index (ALI)	65.93 (34.84)	66.85 (35.12)	56.23 (30.18)	<.001
Femoral neck BMD, g/cm^2^	0.82 (0.15)	0.84 (0.14)	0.57 (0.08)	<.001
Total femur BMD, g/cm^2^	0.96 (0.16)	0.99 (0.14)	0.70 (0.10)	<.001
Lumbar spine BMD, g/cm^2^	1.03 (0.15)	1.05 (0.13)	0.77 (0.10)	<.001
Femoral neck T-score	−0.44 (1.30)	−0.25 (1.16)	−2.57 (0.72)	<.001
Lumbar spine T-score	−0.22 (1.36)	−0.05 (1.22)	−2.61 (0.91)	<.001

BMD = bone mineral density.

† Mean (SD) for continuous variables; unweighted n (weighted %) for categorical variables.

* *P* values from design-based Kruskal–Wallis tests (continuous) and Rao-Scott adjusted chi-square tests (categorical).

Participants with osteoporosis exhibited a less favorable immunonutritional profile. Both PNI (51.65 ± 5.27 vs 53.50 ± 10.04, *P* < .001) and ALI (56.23 ± 30.18 vs 66.85 ± 35.12, *P* < .001) were lower in the osteoporosis group. They also had lower BMI and waist circumference (25.12 vs 28.06 kg/m^2^; 90.87 vs 96.73 cm; both *P* < .001), higher prevalences of diabetes (11% vs 7%, *P* < .001) and hypertension (42% vs 28%, *P* < .001), and were less likely to be current drinkers (49% vs 70%, *P* < .001), whereas smoking status did not differ significantly between groups (*P* = .347). Collectively, these between-group differences highlight the need for multivariable adjustment and robustness analyses to evaluate associations of PNI and ALI with osteoporosis independent of demographic, socioeconomic, lifestyle, and comorbidity profiles.

### 3.2. Association between immunonutritional indices and osteoporosis

As shown in Table 2, both immunonutritional indices were inversely associated with osteoporosis in survey-weighted logistic regression models, although the strength and consistency of associations differed between PNI and ALI.

**Table 2:** PNI/ALI and osteoporosis (survey-weighted logistic).

Exposure	Level	OR (95% CI)	*P* value	OR (95% CI)	*P* value	OR (95% CI)	*P* value	OR (95% CI)	*P* value
ALI	Per 1-SD increment	**0.628 (0.563–0.700**)	**<.001**	**0.742 (0.664–0.829**)	**<.001**	**0.748 (0.670– 0.835**)	**<.001**	**0.766 (0.686–0.854**)	**<.001**
	Q1 (Ref)	1.00 (Reference)		1.00 (Reference)		1.00 (Reference)		1.00 (Reference)	
	Q2	**0.612 (0.515–0.728**)	**<.001**	**0.750 (0.613–0.919**)	**.006**	**0.746 (0.611–0.911**)	**.005**	**0.766 (0.626–0.939**)	**.011**
	Q3	**0.537 (0.422–0.682**)	**<.001**	**0.701 (0.533–0.922**)	**.012**	0.760 (0.571–1.012)	.060	0.804 (0.605–1.069)	.131
	Q4	**0.385 (0.316–0.468**)	**<.001**	**0.537 (0.420–0.688**)	**<.001**	**0.565 (0.429–0.743**)	**<.001**	**0.590 (0.447–0.778**)	**<.001**
	*P* for trend		**<.001**		**<.001**		**<.001**		**.001**
PNI	Per 1-SD increment	**0.439 (0.369–0.523**)	**<.001**	**0.852 (0.726–0.999**)	**.048**	**0.773 (0.641–0.931**)	**.007**	**0.782 (0.648–0.944**)	**.011**
	Q1 (Ref)	1.00 (Reference)		1.00 (Reference)		1.00 (Reference)		1.00 (Reference)	
	Q2	**0.548 (0.457–0.656**)	**<.001**	**0.762 (0.623–0.930**)	**.008**	**0.756 (0.603–0.949**)	**.016**	**0.766 (0.610–0.962**)	**.022**
	Q3	**0.482 (0.389–0.597**)	**<.001**	0.892 (0.698–1.140)	.355	0.908 (0.679–1.212)	.507	0.920 (0.683–1.240)	.581
	Q4	**0.394 (0.317–0.489**)	**<.001**	0.904 (0.708–1.154)	.411	0.814 (0.610–1.087)	.161	0.833 (0.625–1.111)	.210
	*P* for trend		**<.001**		.439		.235		.314

Models: Crude (unadjusted); Model 1 (age, sex, race); and Model 2 (+education, PIR, marital). Model 3 (+smoking, alcohol, diabetes, hypertension, creatinine; +BMI except ALI to avoid overadjustment). Bold indicates *P* < .05.

ALI = Advanced Lung Cancer Inflammation Index, BMI = Body mass index, CI = confidence interval, OR = odds ratio, PNI = Prognostic Nutritional Index, SD = standard deviation.

For PNI, each 1-SD increase was associated with lower odds of osteoporosis in the fully adjusted model (OR 0.782, 95% CI 0.648–0.944; *P* = .011). However, when analyzed by quartiles, the association was attenuated after full adjustment: compared with Q1, only Q2 remained statistically significant (OR 0.766, 95% CI 0.610–0.962; *P* = .022), whereas Q3 and Q4 were not (Q4 OR 0.833, 95% CI 0.625–1.111; *P* = .210). The linear trend across quartiles was not significant (*P* for trend = .314).

In contrast, ALI showed a more consistent inverse association across specifications. Each 1-SD increase in ALI was associated with lower odds of osteoporosis in the fully adjusted model (OR 0.766, 95% CI 0.686–0.854; *P* < .001). In quartile analyses, higher ALI levels were generally associated with lower odds of osteoporosis, with the strongest association observed in Q4 versus Q1 (OR 0.590, 95% CI 0.447–0.778; *P* < .001), and a significant trend across quartiles (*P* for trend = .001).

### 3.3. Nonlinear relationship analysis

**Figure 2.**
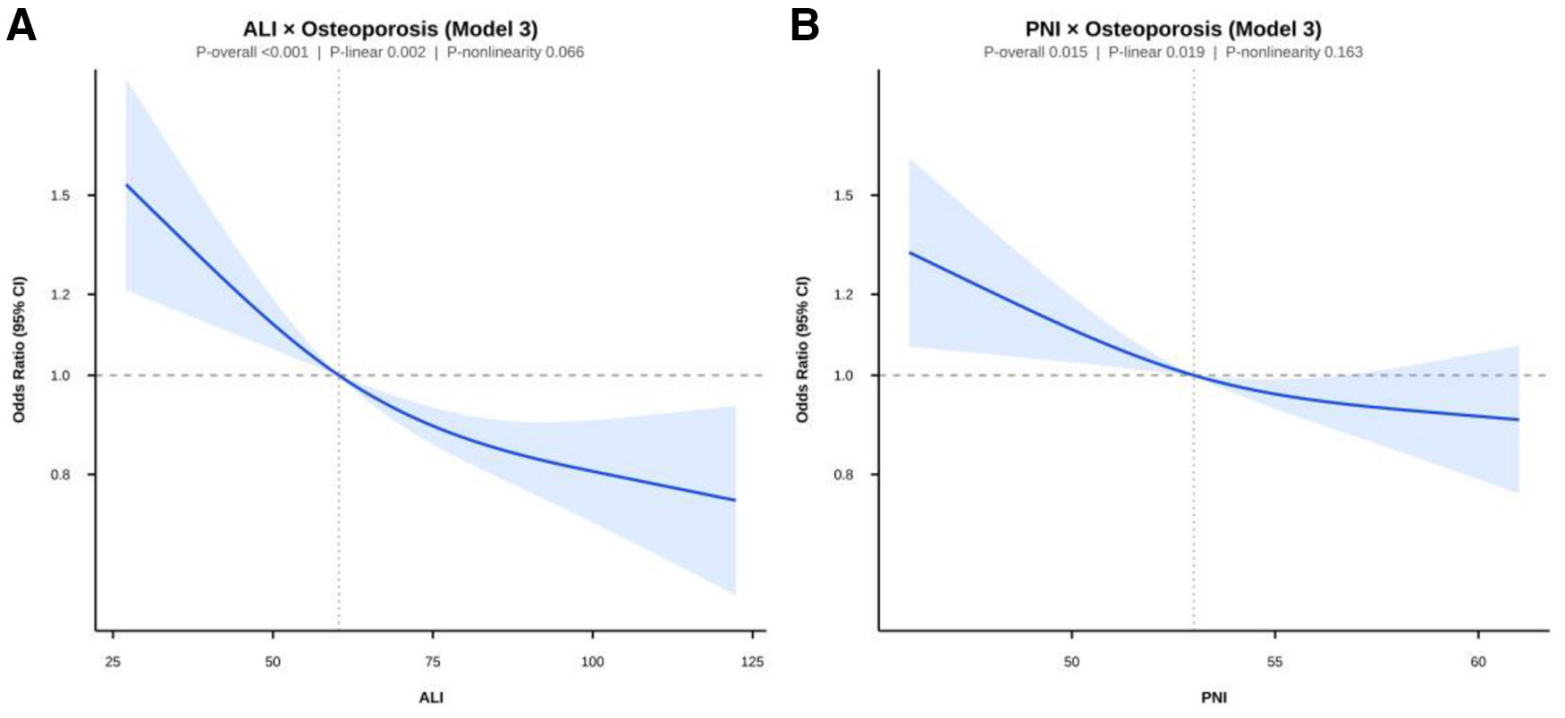
Restricted cubic spline analyses of the associations between ALI (A) and PNI (B) and osteoporosis (ORs with 95% CIs) in Model 3, with *P* values for overall association, linearity, and nonlinearity reported. ALI = Advanced Lung Cancer Inflammation Index, CI = confidence interval, OR = odds ratio, PNI = Prognostic Nutritional Index.

Restricted cubic spline analyses (Fig. 2) were used to characterize the dose–response patterns between the immunonutritional indices and osteoporosis. For ALI (Fig. 2A), the odds of osteoporosis decreased as ALI increased (*P*-overall < .001; *P*-linear = .002), and there was no strong evidence of departure from linearity (*P*-nonlinearity = .066). Visually, the decline appeared steeper at lower ALI values and then became more gradual across the higher range.

For PNI (Fig. 2B), the spline curve suggested an overall inverse association (*P*-overall = .015; *P*-linear = .019), with no evidence of nonlinearity (*P*-nonlinearity = .163). The curve also indicated that the inverse association weakened at higher PNI levels, consistent with an apparent attenuation/plateau toward the upper range.

### 3.4. Subgroup and interaction analyses

**Figure 3.**
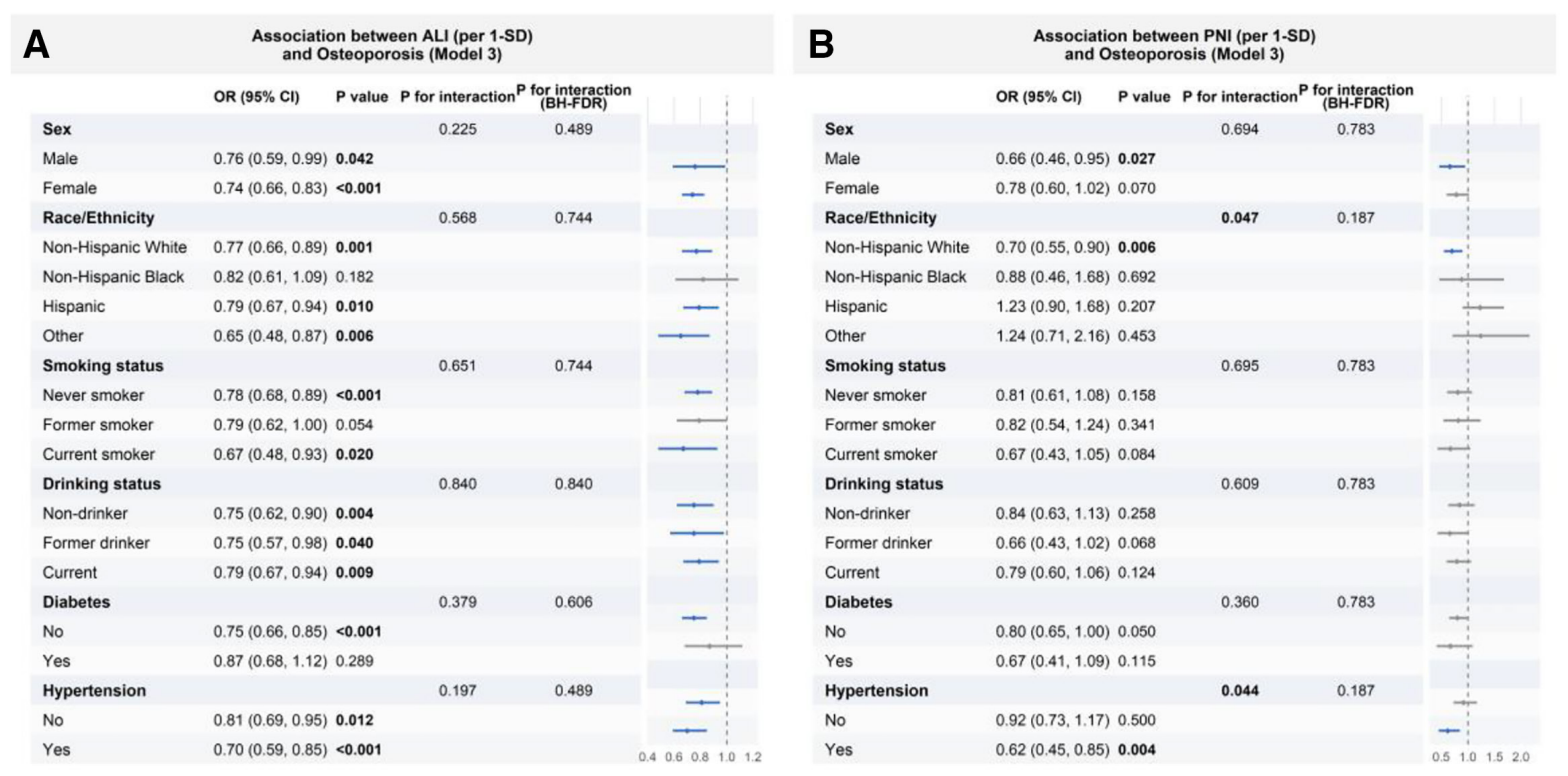
Subgroup analyses of the associations between ALI (A) and PNI (B) per 1-SD increase and osteoporosis (ORs with 95% CIs) in Model 3, with interaction *P* values and BH-FDR-adjusted interaction *P* values reported. ALI = Advanced Lung Cancer Inflammation Index, BH-FDR = Benjamini–Hochberg false discovery rate, CI = confidence interval, OR = odds ratio, PNI = Prognostic Nutritional Index, SD = standard deviation.

Survey-weighted subgroup analyses based on Model 3 are shown in Figure 3A. The inverse association of ALI with osteoporosis was directionally consistent across all prespecified strata, with subgroup-specific ORs uniformly below 1.0 (approximately 0.65–0.87). Although the association appeared more pronounced in some subgroups (e.g., current smokers and participants without diabetes), formal interaction testing did not support effect modification on the multiplicative scale (all *P* for interaction ≥ .197; all Benjamini–Hochberg false discovery rate [BH-FDR]-adjusted *P* ≥ .489), indicating broadly comparable associations across sex, race/ethnicity, smoking, alcohol consumption, diabetes, and hypertension.

In contrast, subgroup estimates for PNI (Fig. 3B) were more heterogeneous. An inverse association was evident in several strata (e.g., men, non-Hispanic White participants, and those with hypertension), whereas estimates were closer to null or exceeded 1.0 in others, suggesting less stable subgroup performance. Nominal interaction signals were observed for race/ethnicity (*P* for interaction = .047) and hypertension (*P* for interaction = .044); however, neither remained significant after BH-FDR adjustment (both BH-FDR = 0.187). Given multiple testing and limited precision in smaller subgroups, PNI subgroup patterns should be interpreted as exploratory and hypothesis-generating rather than confirmatory.

### 3.5. Sensitivity analyses

**Figure 4.**
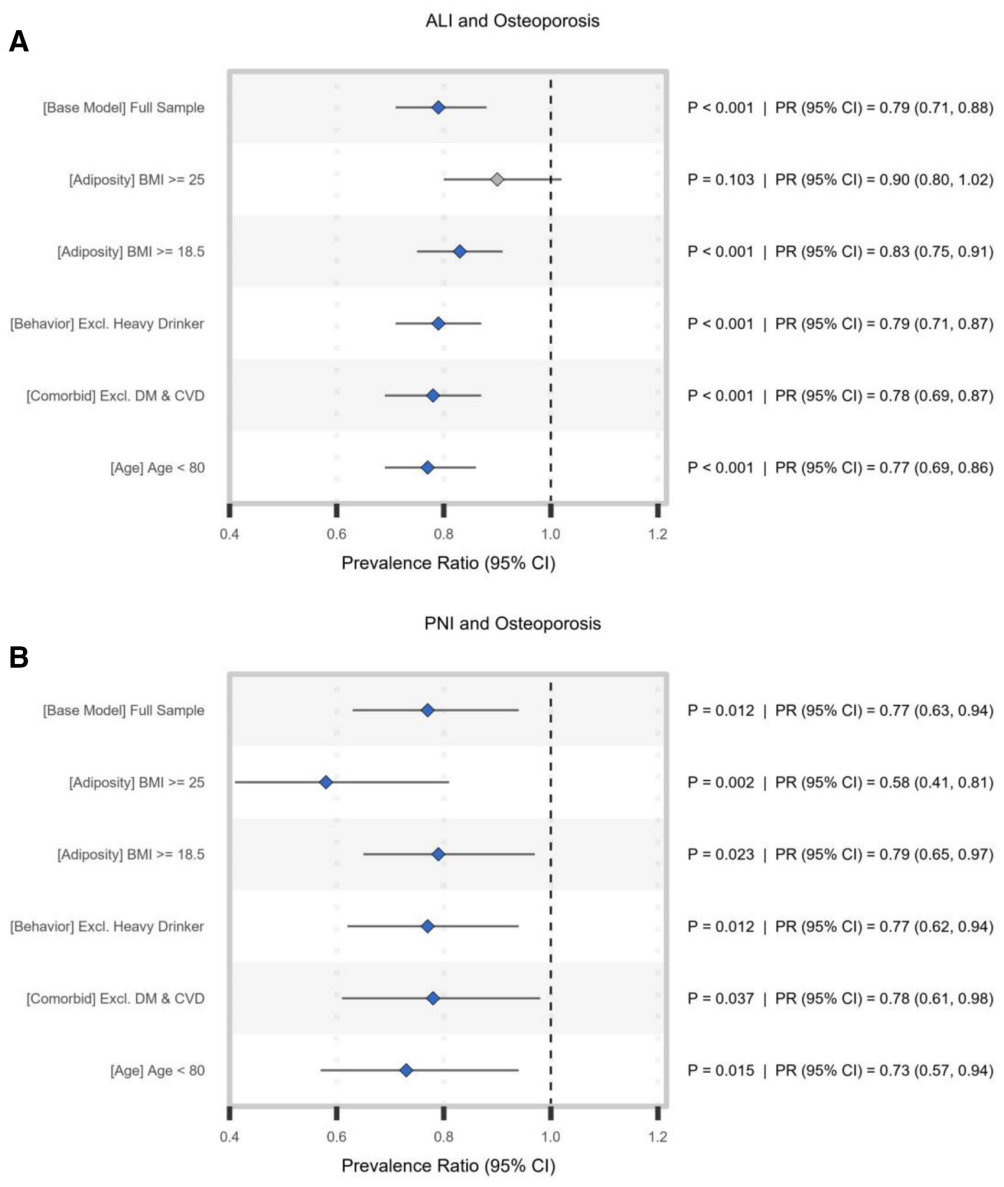
Sensitivity analyses of the associations between ALI (A) and PNI (B) and osteoporosis across prespecified analytic scenarios (PRs with 95% CIs) in the fully adjusted model, with corresponding *P* values reported. ALI = Advanced Lung Cancer Inflammation Index, BMI = body mass index, CI = confidence interval, PNI = Prognostic Nutritional Index, PR = prevalence ratio.

We first conducted prespecified scenario-based sensitivity analyses to assess consistency under alternative analytic restrictions (Fig. 4). In the fully adjusted PR analyses, ALI remained inversely associated with osteoporosis in the full sample (PR 0.79, 95% CI 0.71–0.88) and most restrictions, but attenuated and became nonsignificant when restricting to BMI ≥ 25 (PR 0.90, 95% CI 0.80–1.02; *P* = .103). PNI showed directionally inverse PRs across scenarios, with the strongest association in the BMI ≥ 25 restriction (PR 0.58, 95% CI 0.41–0.81).

As a complementary check for covariate imbalance, we constructed CBW and confirmed improved balance by |SMD| (Supplementary Fig. S1, Supplemental Digital Content 1). Under CBW, ALI remained statistically significant and consistent across estimators (weighted-only OR 0.83, 95% CI 0.69–0.98; *P* = .032; doubly robust OR 0.79, 95% CI 0.64–0.97; *P* = .027), comparable to the traditional adjusted model (OR 0.80, 95% CI 0.65–0.98; *P* = .035; Supplementary Table S1, Supplemental Digital Content 2). In contrast, PNI was not robust under CBW (doubly robust OR 0.94, 95% CI 0.76–1.17; *P* = .580; weighted-only OR 1.13, 95% CI 0.93–1.37; *P* = .223), consistent with the null traditional dichotomized model (OR 0.94, 95% CI 0.77–1.16; *P* = .587; Supplementary Table S1, Supplemental Digital Content 2). Overall, scenario-based PRs supported broadly inverse patterns, whereas CBW-based estimates indicated stronger cross-estimator robustness for ALI and greater specification sensitivity for PNI. It is important to note that small shifts in point estimates between the main models and sensitivity scenarios reflect differences in effect scales (ORs vs PRs) and modeling specifications, rather than true heterogeneity.

## 4. Discussion

In this large, nationally representative analysis of US adults, we observed that 2 immunonutritional indices – ALI and PNI – were lower among participants with osteoporosis and were inversely associated with osteoporosis prevalence in minimally adjusted models. After comprehensive adjustment, ALI remained more consistently inversely associated with osteoporosis, including an approximately monotonic dose–response pattern and persistence across robustness checks, whereas the association for PNI was attenuated under more stringent estimation strategies. Overall, these findings indicate that ALI showed a more stable cross-sectional association across the analytic approaches used in this study, although this should not be interpreted as evidence of causality or predictive superiority.

### 4.1. Interpretation within an osteoimmunology framework

The immune system and skeleton are tightly interconnected (“osteoimmunology”), and osteoporosis is increasingly understood as the downstream manifestation of chronic low-grade inflammation, impaired anabolic capacity, and reduced physiologic reserve,^[22]^ rather than a purely endocrine or mineral disorder. Both PNI and ALI incorporate serum albumin and lymphocyte count, which plausibly index two core domains relevant to bone remodeling: nutritional substrate/protein–energy status and adaptive immune competence.

Albumin is commonly interpreted as a marker of nutritional and inflammatory status. In clinical and epidemiologic studies, hypoalbuminemia often co-occurs with elevated inflammatory cytokines and catabolic states, which are environments that favor osteoclast activation and impair osteoblast function. Likewise, lymphocyte count – while not a bone-specific biomarker – may reflect immune competence, frailty, and systemic inflammatory burden, all of which have been linked to poorer musculoskeletal outcomes. Importantly, albumin and lymphocytes serve as integrative proxies reflecting multiple upstream processes that jointly influence skeletal homeostasis.

### 4.2. Why ALI showed a more consistent association than PNI: possible physiologic and modeling considerations

A key observation in our study is that ALI retained an inverse association with osteoporosis across modeling strategies, whereas PNI did not. This divergence is biologically and methodologically plausible because ALI embeds two additional domains that are both strongly related to bone health: innate-inflammatory activity (through NLR) and body mass/mechanical loading (through BMI).

First, inflammation captured by NLR may be closer to the pathophysiology of bone loss than lymphocytes alone. Chronic inflammatory signaling (e.g., IL-6/TNF-related pathways) promotes osteoclastogenesis and bone resorption via RANKL-mediated mechanisms while suppressing bone formation.^[23]^ NLR partially reflects the balance between innate inflammatory activation (neutrophil predominance) and adaptive immune capacity (lymphocytes).^[24]^ By incorporating NLR, ALI may more directly reflect the inflammatory milieu that accelerates skeletal catabolism, which PNI – dominated by albumin and lymphocytes – may capture only indirectly.

Second, BMI represents the mechanical and body-composition dimension of bone health. Mechanical loading is among the strongest determinants of BMD, and low body mass is a well-established risk factor for osteoporosis. Because BMI is explicitly included in ALI, this index intrinsically integrates a powerful musculoskeletal determinant that is external to PNI. Consistent with this, when BMI (or related adiposity measures) is added to traditional regression models for PNI, the independent signal of PNI may diminish – either because BMI is a confounder strongly correlated with nutritional status, because BMI mediates part of the pathway from poor nutritional reserve to reduced bone mass, or both. From this perspective, ALI can be interpreted as a composite that summarizes mechanical loading/body reserve (BMI), nutritional/inflammatory reserve (albumin), and inflammatory imbalance (NLR). Such multi-domain integration may partly explain why ALI showed a more consistent association than PNI in our analyses.

The subgroup analyses also provide additional context for interpreting these findings. The association between ALI and osteoporosis appeared evident among women, which is clinically plausible because women, particularly after menopause, are more susceptible to accelerated bone loss and osteoporosis. Nevertheless, this sex-related pattern should be interpreted cautiously, as the interaction analysis did not support a firm conclusion of sex-specific effect modification. Other covariates may also have influenced the observed associations. Age and sex are closely related to baseline osteoporosis risk, whereas race/ethnicity, smoking, alcohol consumption, diabetes, hypertension, and body composition may shape both inflammatory–nutritional status and skeletal health. Therefore, these factors may act as important confounders or clinical contexts in which the associations of ALI and PNI with osteoporosis are expressed. In contrast to ALI, the subgroup estimates for PNI were less stable, suggesting that PNI may be more sensitive to differences in population characteristics and covariate distribution. Overall, these subgroup findings should be regarded as exploratory and require confirmation in future studies.

### 4.3. Robustness checks and what they do (and do not) prove

We further evaluated robustness using CBW, which improved covariate balance compared with the raw survey-weighted sample. Under CBW-based estimation, the inverse association of ALI with osteoporosis remained directionally consistent and statistically significant across estimators, whereas PNI remained null. These results suggest that the inverse association for ALI was more stable across the prespecified modeling approaches examined, although residual bias and model dependence cannot be excluded.

However, CBW does not transform cross-sectional observational data into causal evidence. Even with excellent balance on measured covariates, estimates can still be influenced by unmeasured confounding, measurement error, and violations of positivity (limited overlap), and weighting can increase variance in some settings.^[25]^ Accordingly, the CBW findings should be interpreted as robustness to measured confounding patterns, not as proof of causality.

### 4.4. Clinical and public-health implications

From a practical standpoint, ALI can be derived from routinely available measurements (height/weight and a standard blood count with albumin). In settings where DXA is not readily accessible, these findings support further interest in ALI as a simple adjunctive indicator linked to bone health, rather than a substitute for formal bone density assessment.^[26]^ At the same time, findings from prior NHANES analyses conducted in selected disease-specific subpopulations (for example, adults with type 2 diabetes) may not generalize directly to the broader US adult population, which underscores the value of population-wide comparative analyses.

More broadly, our findings reinforce the view that osteoporosis risk is shaped not only by calcium/vitamin D and hormones, but also by body reserve and chronic inflammatory burden.^[27]^ Still, it would be premature to claim that “improving ALI” itself prevents osteoporosis. Interventions should instead target plausible upstream levers – nutritional adequacy, sarcopenia prevention, healthy weight maintenance, and inflammatory disease control – and future prospective studies should test whether ALI meaningfully improves risk prediction beyond established clinical variables.

### 4.5. Strengths and limitations

This study has several strengths, including the use of a large, nationally representative dataset and a prespecified analytic strategy that incorporated comprehensive covariate adjustment, nonlinearity assessment, subgroup evaluation, and robustness checks using covariate balancing weights. These features collectively reduce the likelihood that the observed ALI association is driven solely by a single modeling choice.

Limitations should be emphasized. First, the cross-sectional design precludes temporal inference; reverse causation is plausible (e.g., osteoporosis-associated frailty leading to weight loss and inflammatory dysregulation, thereby lowering ALI).^[28]^ Second, residual confounding remains possible, including factors not fully captured in our models such as lifetime physical activity, detailed dietary patterns and supplementation, medication use (e.g., glucocorticoids), menopausal status and hormone therapy, comorbidity severity, and inflammatory conditions. In addition, osteoporosis was defined using *T*-scores based on young White women aged 20 to 29 years as the reference population, which may not fully account for sex- and race/ethnicity-related differences in peak bone mass. Third, both PNI and ALI are composite indices developed for other clinical contexts, and their optimal thresholds and predictive utility for skeletal outcomes require external validation. Finally, because BMI is embedded within ALI and was therefore not additionally adjusted for in ALI models to avoid component overadjustment, the ALI and PNI models are structurally asymmetric. Accordingly, direct comparisons of effect magnitudes between ALI and PNI should be interpreted cautiously, and the stronger association observed for ALI may partly reflect the established BMI–BMD relationship. Component-wise and mediation-oriented analyses would clarify mechanism and improve interpretability.

### 4.6. Future directions

Future longitudinal studies should prioritize designs that can establish temporality and reduce reverse causation, including prospective cohorts with repeated measurements of ALI/PNI and bone outcomes, as well as analyses incorporating incident osteoporosis, fragility fractures, and time-to-event endpoints.^[29]^ In addition to confirming associations, subsequent work should quantify the incremental predictive value of ALI beyond conventional risk factors and established fracture risk tools (e.g., FRAX), using discrimination, calibration, reclassification metrics, and decision-curve analysis to evaluate clinical utility. Mechanistic clarification is also warranted: component-wise and mediation-oriented analyses should disentangle the relative contributions of BMI, albumin, and NLR, and assess whether ALI captures nutritional reserve, systemic inflammation, or both, including potential effect modification by age, sex/menopausal status, obesity phenotypes, and comorbid cardiometabolic conditions. Finally, interventional and quasi-experimental studies should examine whether improving the underlying domains reflected by ALI – such as nutritional optimization, inflammation reduction, and maintenance of healthy body composition – corresponds to meaningful improvements in BMD trajectories and reductions in osteoporosis and fracture risk, which may help clarify the clinical interpretability of ALI.

## 5. Conclusion

In this nationally representative sample of US adults, higher ALI was consistently associated with lower odds of prevalent osteoporosis across multivariable models, dose–response analyses, and CBW-based robustness checks, whereas PNI showed weaker and less stable associations. As a composite integrating BMI, albumin, and NLR, ALI may warrant further evaluation as a marker associated with bone health. Given the cross-sectional design, these findings should be interpreted as associational rather than causal.

## Author contributions

**Conceptualization:** Pujian Zhong, Zhuoqin Pan.

**Data curation:** Pujian Zhong.

**Formal analysis:** Wenren Wu.

**Funding acquisition:** Wenren Wu, Zhuoqin Pan.

**Investigation:** Chaoyi Yin.

**Methodology:** Chaoyi Yin.

**Project administration:** Binshan Zhang, Zhuoqin Pan.

**Resources:** Binshan Zhang, Zhiqing Chen, Zhuoqin Pan.

**Supervision:** Yiqi Wu, Zhuoqin Pan.

**Validation:** Yiqi Wu, Canwei Hu.

**Visualization:** Canwei Hu.

**Writing – original draft:** Pujian Zhong.

**Writing – review & editing:** Zhiqing Chen, Wenren Wu, Zhuoqin Pan.




