# Supplementary material for: Comparative associations of the Advanced Lung Cancer Inflammation Index and Prognostic Nutritional Index with osteoporosis among adults in the United States: A cross-sectional analysis
Source: Medicine (Baltimore). 2026 Jul 17;105(29):e49531. doi: 10.1097/MD.0000000000049531 (PMC13384710; doi:10.1097/MD.0000000000049531)
Supplement: Supplementary file 1 [file medi-105-e49531-s001.pdf]

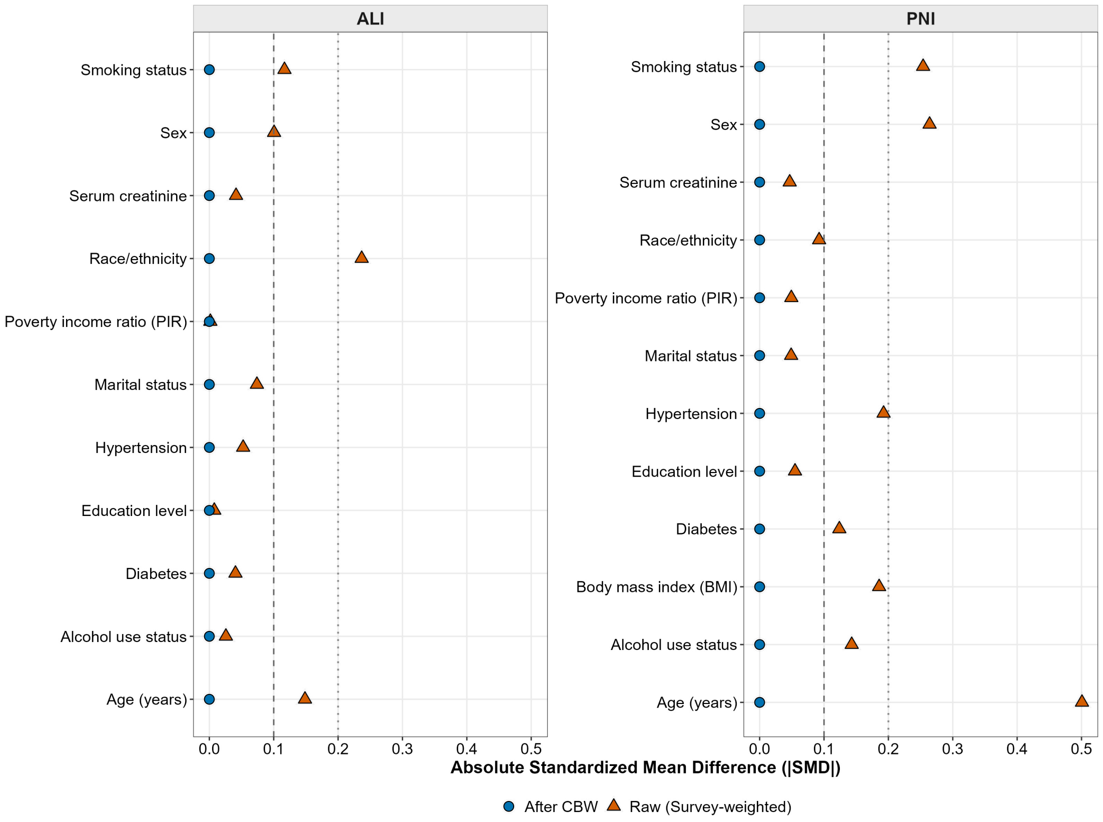

Supplementary Figure S1 (A–B). Covariate balance before and after covariate balancing weighting (CBW) for the ALI (A) and PNI (B) analyses, assessed by absolute standardized mean differences (|SMD|) for raw survey-weighted and CBW-weighted samples.
