# Supplementary material for: Comparative associations of the Advanced Lung Cancer Inflammation Index and Prognostic Nutritional Index with osteoporosis among adults in the United States: A cross-sectional analysis
Source: Medicine (Baltimore). 2026 Jul 17;105(29):e49531. doi: 10.1097/MD.0000000000049531 (PMC13384710; doi:10.1097/MD.0000000000049531)
Supplement: Supplementary file 2 [file medi-105-e49531-s002.docx]

Supplementary Table S1. Sensitivity Analysis (Calibration-based Balancing Weights)

| **Index** | **Analysis Model** | **OR (95% CI)** | **P Value** |  |
| --- | --- | --- | --- | --- |
| ALI | CBW (Logit calibration) — Doubly Robust | 0.79 (0.64, 0.97) | 0.027 | * |
|  | CBW (Logit calibration) — Weighted only | 0.83 (0.69, 0.98) | 0.032 | * |
|  | Traditional (Binary split at weighted median; Survey-weighted + Adjusted) | 0.80 (0.65, 0.98) | 0.035 | * |
| PNI | CBW (Logit calibration) — Doubly Robust | 0.94 (0.76, 1.17) | 0.580 |  |
|  | CBW (Logit calibration) — Weighted only | 1.13 (0.93, 1.37) | 0.223 |  |
|  | Traditional (Binary split at weighted median; Survey-weighted + Adjusted) | 0.94 (0.77, 1.16) | 0.587 |  |
| Note: We used calibration-based balancing weights (CBW) via survey::calibrate (logit calibration with bounds). | | | | |
